# Cerebral autoregulation is heterogeneous in different stroke mechanism of ischemic stroke caused by intracranial atherosclerotic stenosis

**DOI:** 10.1002/brb3.1907

**Published:** 2020-10-23

**Authors:** Ge Tian, Zhong Ji, Zhenzhou Lin, Suyue Pan, Jia Yin

**Affiliations:** ^1^ Department of Neurology Nanfang Hospital Southern Medical University Guangzhou China

**Keywords:** Cerebral autoregulation, intracranial atherosclerotic stenosis, ischemic stroke, stroke mechanism

## Abstract

**Background and Purpose:**

Intracranial atherosclerotic stenosis (ICAS) is the most common cause of ischemic stroke (IS) and is associated with stroke recurrence. It results in IS due to a variety of mechanisms. However, the influence of brain reserve mechanism on different stroke mechanism is still unclear. Cerebral autoregulation (CA) is an important brain reserve mechanism and is impaired after IS. This study aimed to explore the impaired pattern of CA and assess the association between CA and stroke risk factors in different stroke mechanism caused by ICAS.

**Methods:**

IS patients with ICAS (50%–99% stenosis/occlusion) in middle cerebral artery (MCA) or internal carotid artery were enrolled to receive CA examinations within 7 days after onset. Healthy volunteers were also recruited as controls. CA was recorded from spontaneous fluctuations of blood pressure and MCA flow velocity. Transfer function analysis was used to derive CA parameters, including phase difference (PD) and coherence in the low‐frequency range (0.06–0.12 Hz).

**Results:**

A total of 89 IS patients and 90 healthy controls were included. Compared with controls, CA was impaired ipsilaterally in patients with parent artery atherosclerosis occluding penetrating artery (POPA) while CA was bilaterally impaired in other stroke mechanisms. And CA on ipsilateral hemisphere was correlated with hypertension/hyperlipidemia in patients with POPA (*r* = −0.481, *p* = .008; *r* = −0.484, *p* = .008). While CA on ipsilateral hemisphere was correlated with perfusion parameter including the arterial spin‐labeling (ASL) parameter cerebral blood flow (CBF) (*r* = 0.893, *p* = .007) and collateral circulation status the American Society of Interventional and Therapeutic Neuroradiology/Society of Interventional Radiology (ASITN/SIR) (*r* = 0.610, *p* = .021) in patients with hypoperfusion mechanism.

**Conclusion:**

In IS patients, CA was impaired heterogeneously and was correlated with different risk factors in varied stroke mechanism. CA can be as an informative determinant of stroke risk in patients with ICAS and to help improving individualized treatment strategies in the presence of ischemic stroke caused by ICAS.

## Graphical abstract

Currently, there still exists debates on impairment pattern of cerebral autoregulation (CA) in patients with intracranial atherosclerotic stenosis (ICAS). Our study found that CA was impaired ipsilaterally in patients with parent artery atherosclerosis occluding penetrating artery (POPA) while CA was bilaterally impaired in other stroke mechanisms. And CA on ipsilateral hemisphere was correlated with hypertension/hyperlipidemia in patients with POPA, while CA on ipsilateral hemisphere was correlated with perfusion parameter and collateral circulation status in patients with hypoperfusion mechanism. The finding may suggested that CA can be as an informative determinant of stroke risk in patients with ICAS and to help improving individualized treatment strategies in the presence of ischemic stroke caused by ICAS.

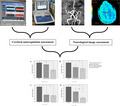

## 1 INTRODUCTION

Intracranial atherosclerotic stenosis (ICAS) is an important cause of ischemic stroke (IS) especially in Asian countries Wityk et al. (1996). In the Chinese population, the incidence of ICAS is up to 46.6% in stroke patients Wang et al. (2014). Furthermore, patients with symptomatic ICAS are also at a high risk of stroke recurrence, up to 25%–30% in two years after an index stroke (Kasner et al., 2006; Mazighi et al., 2006; Wong & Li, 2003). Despite the high prevalence of this high‐risk disease, controversy exists regarding treatment of symptomatic ICAS (sICAS) patients. Previous large clinical trials of ICAS, such as the Warfarin Aspirin Symptomatic Intracranial Disease trial (WASID) and the Stenting and Aggressive Medical Management for Preventing Recurrent Stroke in Intracranial Stenosis trial (SAMMPRIS), have evaluated the effectiveness of thromboembolism prevention and hemodynamic restoration in stroke patients caused by ICAS (Chimowitz et al., 2005; Chimowitz et al., 2011). They both emphasized that treatment effects may differ among the different stroke mechanism of ICAS patients. Therefore, the treatment strategies might be selected based on various aspects including cerebral hemodynamics, plaque components, morphology, and other factors (Feng et al., 2019).

Cerebral autoregulation (CA) is a key mechanism to maintain stable hemodynamic station through ensuring relatively constant cerebral blood flow (CBF) despite fluctuations in arterial blood pressure (ABP) or cerebral perfusion pressure (Beek et al., 2008). It is considered as an important cerebrovascular reserve capacity. We use transfer function analysis (TFA) to get CA parameters including coherence and phase difference (PD) (Claassen et al., 2016). According to previous report, a phase shift of 0° indicates total absence of autoregulation, while a large positive phase shift of 40°–70° can be regarded as intact autoregulation (Gong et al., 2013). The understanding of the pattern of CA in different stroke mechanism of ICAS may help in stroke prevention, treatment, and outcome predictions. However, previous studies provided some conflicting results about CA impaired pattern in stroke. Some scientific data regarded that CA was impaired focally in the affected hemisphere while some showed bilaterally impaired (Aries et al., 2010; Reinhard et al., 2008; Xiong et al., 2017).

We hypothesis that in ischemic stroke caused by ICAS, the impaired pattern of CA is heterogeneous among different stroke mechanisms and is correlated with different stroke risk factors. In the present study, we attempt to investigate this hypothesis by assessing the CA in ischemic stroke patients caused by ICAS using TFA.

## 2 METHODS

### 2.1 Participants

The study was approved by the Medical Ethics Committee of Nanfang Hospital. All subjects have signed a written informed consent before entering the study. We carried out a prospective study of consecutive admissions to the Department of Neurology at the Nanfang Hospital during November 2017 to September 2019.

We prospectively examined a cohort of consecutive patients with acute ischemic stroke attributed to 50% to 99% intracranial atherosclerotic stenosis in anterior circulation. Patients were included in this study if they (a) ischemic stroke patients admitted within 7 days after symptom onset; (b) the patient underwent diffusion‐weight magnetic resonance imaging (DWI) within 7 days after stroke onset, and computed tomography angiography (CTA) and/or Digital Subtraction Angiography (DSA) within 14 days; (c) the ischemic stroke was attributed to 50%–99% atherosclerotic stenosis of intracranial portion of internal carotid artery (ICA) or middle cerebral artery (MCA) stem as revealed by CTA or DSA; (d) without contralateral intracranial and extracranial major vascular 30%–99% stenosis/occlusion which was confirmed by CTA; (e) had a sufficient bilateral temporal bone window for insonation of MCA. Exclusion criteria included those who (a) had IS attributed to nonatherosclerotic intracranial stenosis; (b) clinical‐DWI correlations which did not match the manifestations of acute infarction; (c) lesions in posterior circulations; (d) had strokes of other determined etiologies, evidence of others mechanisms of arterial wall damage (i.e., vasculitis, Moya‐Moya disease, clot); (e) evidence of cardiac sources of emboli based on transesophageal echocardiography for aortic arch atheroma and patent foramen; (f) received intravenous thrombolysis and/or endovascular therapy; (g) were diagnosed as cancer or mental diseases.

We set up a healthy volunteers database who attended the annual physical examination in Nanfang Hospital from March 2017 to May 2017. In this study, we recruited healthy controls who were age‐matched with AIS patients from the healthy volunteers' database. They also should meet the following inclusion criteria: (a) without intracranial and extracranial vascular stenosis by transcranial Doppler sonography (TCD), carotid artery color Doppler (CD) examination; (b) had a sufficient bilateral temporal bone window for insonation of MCAs; (c) the absence of atrial fibrillation, hyperlipidemia, hypertension, diabetes mellitus, and cerebral vascular disease history; (d) without a history of chronic physical or mental diseases, without an infectious disease in the past month, without a history of smoking or heavy drinking, no being pregnant or lactating.

Collected clinical information included age, gender, history of hypertension, diabetes mellitus and hyperlipidemia, current smoking, previous history of stroke and ischemic heart disease, atrial fibrillation, valvular heart disease, heavy alcohol consumption, National Institute of Health stroke scale (NIHSS) at admission, degree of stenosis, results of neuroimaging, carotid duplex ultrasonography, 24 hr electrocardiography (Holter), and electrocardiography.

Medical management includes antiplatelet therapy Clopidogrel (75 mg) and Aspirin (100 mg), and intensive risk factor management.

## 3 STROKE MECHANISMS, INFARCT PATTERNS ASSESSMENTS, AND STENOSIS DEGREE EVALUATION

Two neurologists and one neuroradiologist were independently reviewed the cases, who were blinded to the clinical data, DWI lesion patterns, CTA, and DSA data. A third reader's opinion was obtained in cases of disagreement. All MRI examinations were performed with a 3 Tesla MR imager (Achieva TX; Philips Healthcare, Netherland). And CTA was performed on a 64‐slice CT scanner (Philips Healthcare, The Netherlands).

### 3.1 Stroke mechanisms were classified as follows

1. Parent artery atherosclerosis occluding penetrating artery (POPA) is in the presence of subcortical lesions in the distribution of perforating vessels that originate at the site of stenosis. In the pattern, infarcts are localized to an area adjacent to the stenosed vessel, which is attributable to the occlusion of the orifice of ≥1 perforators that supply the regions presumably associated with local thrombus formation.
2. Artery‐to‐Artery Embolism. DWI demonstrates infarcts distal to the stenosed vessel in the territory of the relevant artery in artery‐to‐artery embolism. They are usually multiple, scattered, and often associated with perfusion deficits throughout the territory of the stenosed vessel.
3. Hypoperfusion. With hemodynamic impairment, the infarcts are located in borderzone areas, usually are linear in shape and are associated with perfusion deficits distal to the severely stenosed or occluded vessel. Borderzone pattern in the presence of one or more lesions in the internal borderzone region and/or in the cortical borderzone region.
4. Mixed pattern when a combination of any of the previous patterns was present (Lopez‐Cancio et al., 2014; Tatu et al., 1998).

**Figure 1:**
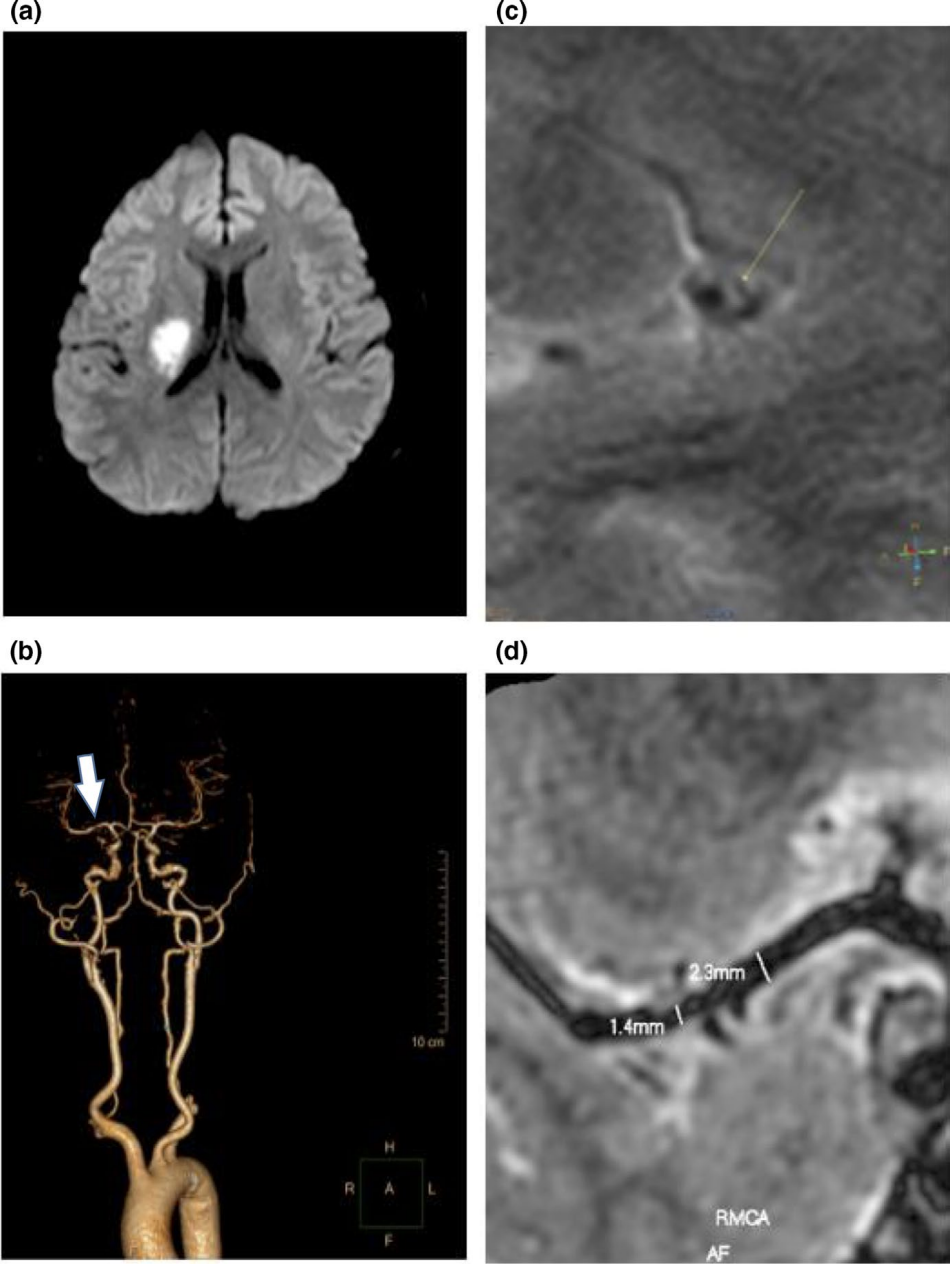
Parent artery atherosclerosis occluding penetrating artery (POPA) stroke mechanism in ischemic stroke patients with ICAS. Images from a 50‐year‐old man with acute cerebral infarction in the right basal ganglia. When the patient was admitted to the hospital, the consciousness was clear, presented with the left side of the nasolabial fold became shallow, the left upper limb muscle strength was level 2, the left lower limb muscle strength was level 5‐, and the right limb muscle strength was level 5. NIHSS on admission was 3, and mRS at discharge was 2. (a) An isolated acute infarction in penetrating artery territory indicating probable parent artery atherosclerosis occluding penetrating artery. (b) CTA showed stenosis in M1 segment of right middle cerebral artery. The arrows indicate stenosis location. (c d) High‐resolution magnetic resonance showed plaque in M1 segment of right middle cerebral artery blocks vessels and leading to 60% lumen stenosis

**Figure 2:**
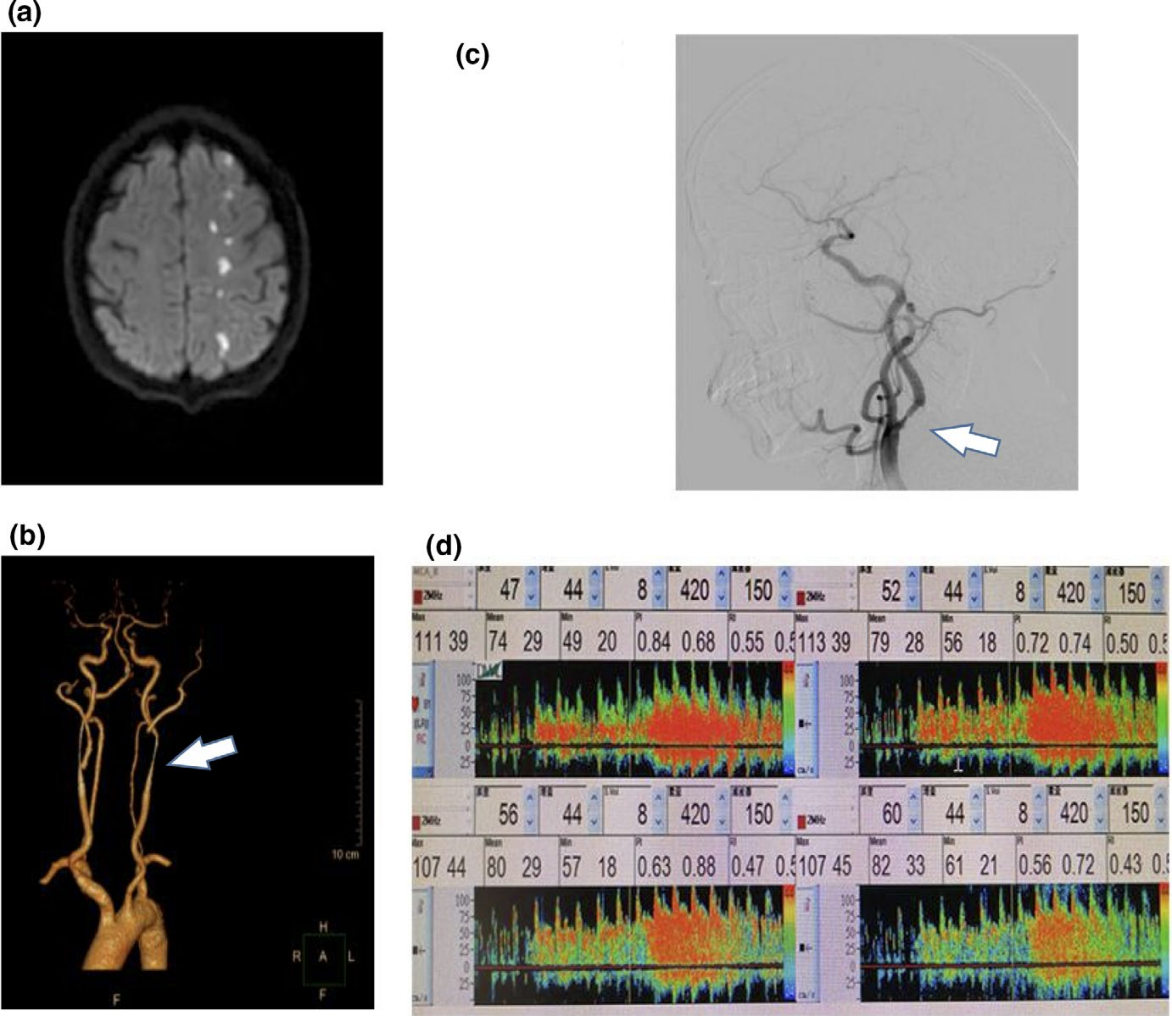
Artery‐to‐Artery embolism stroke mechanism in ischemic stroke patients with ICAS. Images from a 77‐year‐old woman with acute cerebral infarction in the left cerebral hemisphere. When the patient was admitted to the hospital, the consciousness was clear, presented with the right limb muscle strength was level 4, and the left limb muscle strength was level 5. NIHSS on admission was 2, and mRS at discharge was 1. (a) DWI shows small, scattered, cortical embolic infarcts in left hemisphere indicating probable artery‐to‐artery embolism. (b, c) CTA and DSA showed left internal carotid artery sinus ulcer plaque and severe stenosis. The arrows indicate stenosis location. (d) Transcranial doppler ultrasonography can detect symptomatic or asymptomatic embolism during microembolic signal monitoring

**Figure 3:**
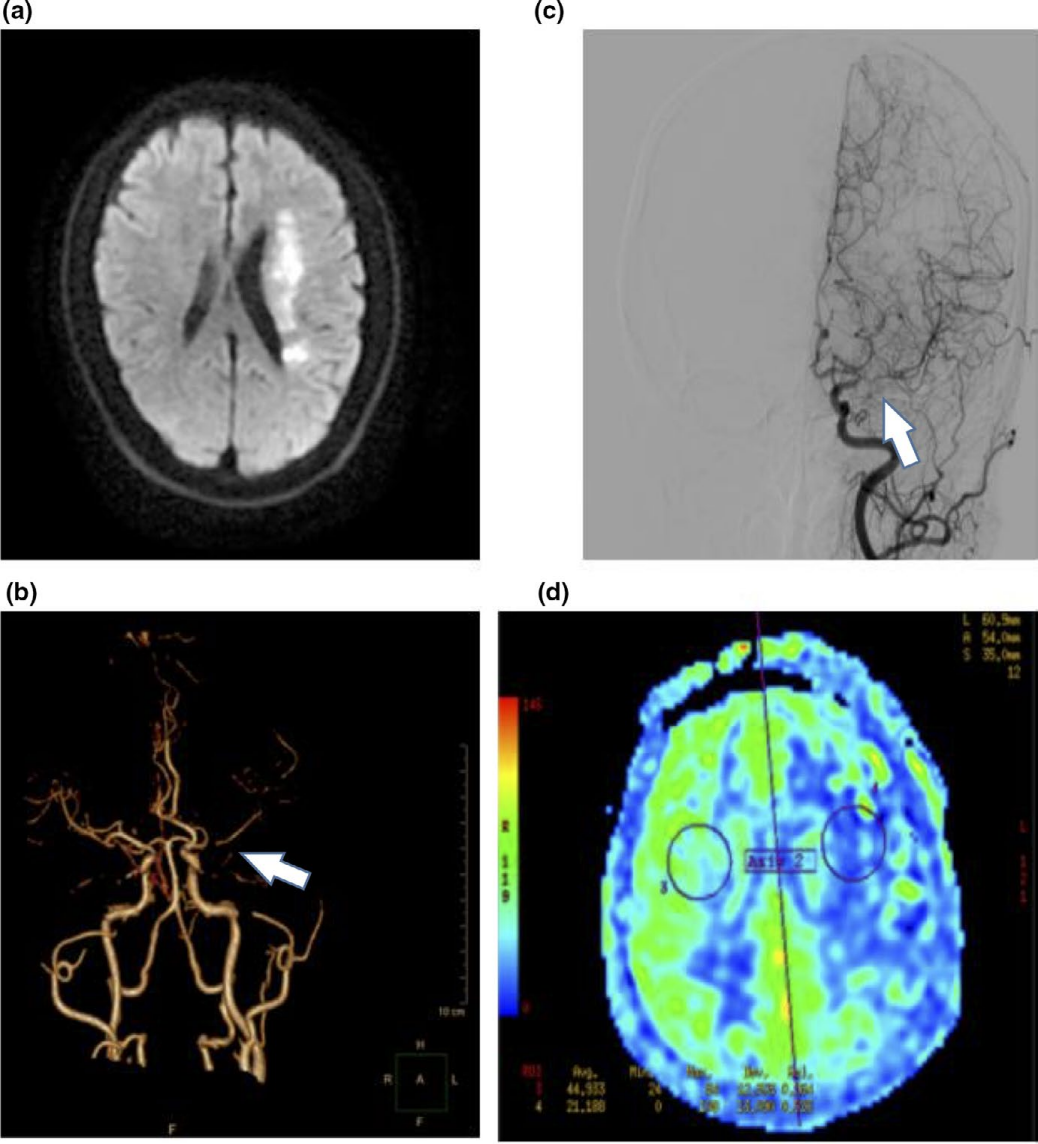
Hypoperfusion stroke mechanism in ischemic stroke patients with ICAS. Images from a 57‐year‐old man with acute cerebral infarction in the left cerebral hemisphere. When the patient was admitted to the hospital, the consciousness was clear, presented with mixed aphasiathe, the right side of the nasolabial fold became shallow, right limb muscle strength was level 0, and the left limb muscle strength was level 5. NIHSS on admission was 19 and mRS at discharge was 4. (a) DWI shows hemodynamic impairment mechanism leading to acute ischemic stroke in left hemisphere. (b,c) CTA and DSA show severe stenosis in left middle cerebral artery. The arrows indicate stenosis location. (d) Arterial spin‐labeling shows hypoperfusion in left hemisphere

**Figure 4:**
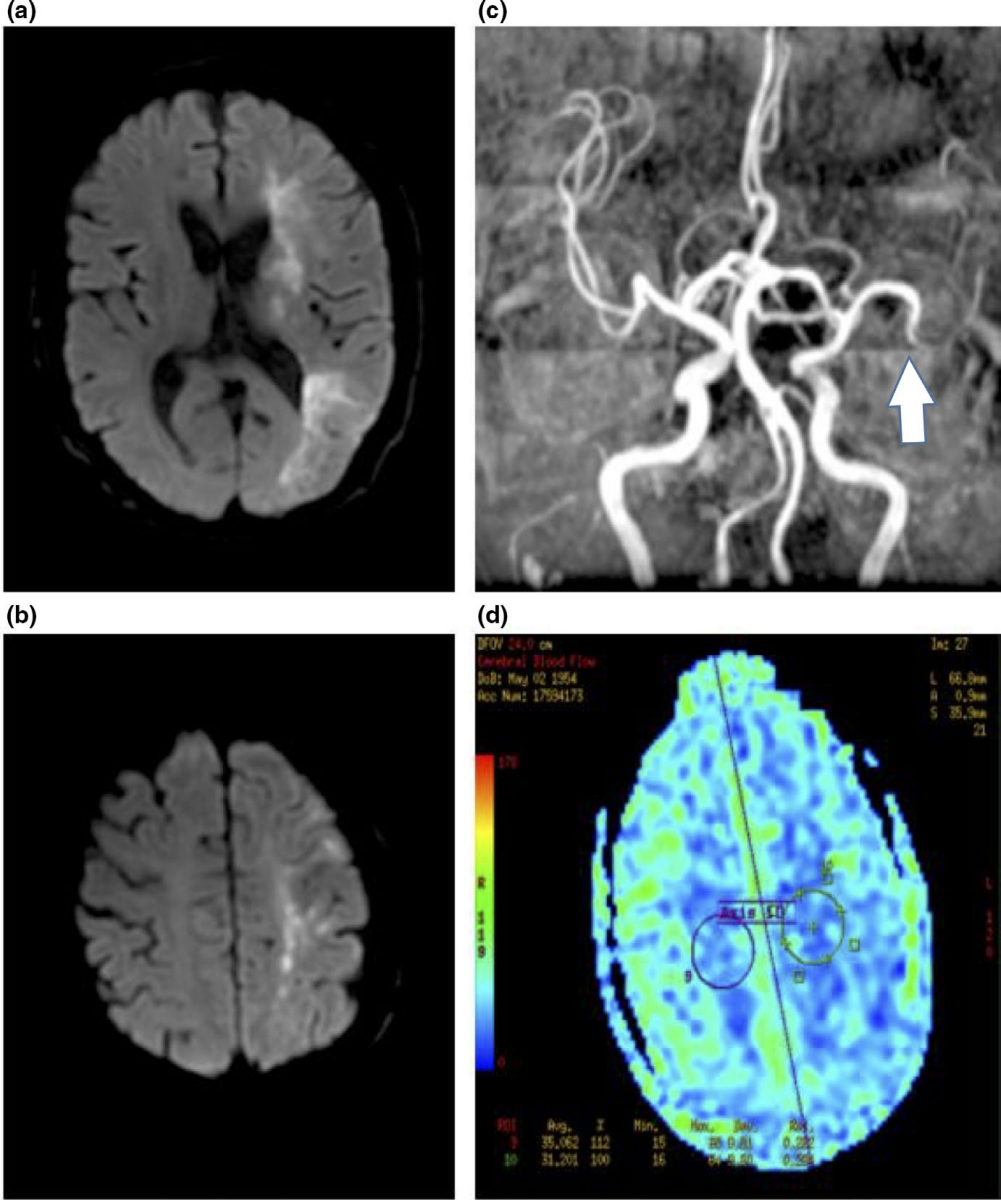
Mixed pattern stroke mechanism in ischemic stroke patients with ICAS. Images from a 64‐year‐old man with acute cerebral infarction in the left cerebral hemisphere. When the patient was admitted to the hospital, the consciousness was clear, presented with mixed aphasiathe, the right side of the nasolabial fold became shallow, right limb muscle strength was level 2, and the left limb muscle strength was level 5. NIHSS on admission was 18, and mRS at discharge was 4. (a,b) Multiple cortical infarctions in DWI indicate mixed mechanism of artery‐to‐artery embolism and hypoperfusion. (c) MRA shows occlusion of left middle cerebral artery. The arrows indicate stenosis location. (d) Arterial spin‐labeling shows hypoperfusion in left hemisphere

All of above were based on Chinese Ischemic Stroke Subclassification system and other previous studies on stroke mechanisms in ICAS (Gao et al., 2011; Qureshi & Caplan, 2014; Figures 1, 2, 3, 4).

### 3.2 Evaluation of collateral circulation, cerebral perfusion, and degree of cerebral artery stenosis

Evaluation of collateral circulation was performed by DSA, utilizing the American Society of Interventional and Therapeutic Neuroradiology/Society of Interventional Radiology (ASITN/SIR) collateral flow grading system (Jagersberg et al., 2010). Grade 0: No collaterals visible adjacent to the ischemic site; Grade 1: Slow collaterals to the periphery of the ischemic site with persistence of some of the defect; Grade 2: Rapid collaterals to the periphery of ischemic site with persistence of some of the defect and to only a portion of the ischemic territory; Grade 3: Collaterals with slow but complete angiographic blood flow of the ischemic bed by the late venous phase; Grade 4: Complete and rapid collateral blood flow to the vascular bed in the entire ischemic territory by retrograde perfusion. Patients with grades 0–2 were grouped as poor collateral status, and grades 3–4 were deemed good collateral status.

Perfusion was assessed using arterial spin‐labeling (ASL) magnetic resonance (MR) imaging.

The degree of arterial stenosis was classified by CTA using warfarin–aspirin symptomatic intracranial disease criteria (Samuels et al., 2000).

### 3.3 Cerebral autoregulation protocol

The CA examination protocol was performed according to the white paper from the International Cerebral Autoregulation Research Network (Claassen et al., 2016). To ensure operational stability, the CA measurement was performed by one fixed person who was blinded to the study design and patients condition. All healthy control subjects were asked to avoid nicotine, caffeine, alcohol, and all kinds of sleep medicines for at least 24 hr before the CA examination. The examination was performed bedside with minimal surrounding stimuli. The control subjects and the patients rested in a supine position with uncrossed legs for more than 15 min before the examination. First, the baseline arterial blood pressure (ABP) was measured at the brachial artery using an automatic blood pressure monitor (Omron 711). Second, we simultaneously recorded continuous spontaneous ABP via a servo‐controlled plethysmograph placed around the left middle finger held at the level of the heart (Finometer Pro, Netherlands) and continuous BFV of MCA at a depth of 45 mm to 60 mm with 2 MHz probes attached to a customized head frame (EMS‐9PB, Shenzhen, China). Meanwhile, the PaCO_2_ level was also monitored, maintaining in stable rang. Data were recorded for 15 min for further data examination analysis. The artifacts were manually removed after recording.

The CA analysis was performed using the multimodal real‐time analysis software ICM + invented by Brain Physics Lab of Cambridge University. According to the continuous ABP signal and bilateral MCA blood flow recordings, autoregulation indices including PD and coherence between the two signal components in the specific frequency domain range (0.008–0.05 Hz) was calculated by the TFA (Guo et al., 2018). We used coherence as a data quality control parameter, and only when the coherence was greater than 0.4, the data were included in the subsequent statistical analysis.

### 3.4 Statistical evaluation

Continuous variables with normal distribution were presented as mean ± standard deviation, and non‐normally distributed continuous variables were presented as median (interquartile range, IQR). Kolmogorov–Smirnov analysis was used to test the normality of data distribution. Frequencies (percentages) were measured for categorical variables. CA data of healthy controls were analyzed based on the mean value of the left and right cerebral sides. To assess intergroup differences, we firstly compared multiple group differences with multiple comparisons (One‐way ANOVA for continuous variable/Kruskal–Wallis rank sum test for categorical variables), if significant, the comparison between the two groups used Student's *t* test (normally distributed), Wilcoxon test (not normally distributed) and Chi‐square or Fisher's exact test as appropriate. The correlation between ICA/MCA stenosis, and autoregulatory parameters (PS, coherency) were assessed by Spearman's rank coefficient method.

## 4 RESULTS

### 4.1 General characteristics

Table 1 summarizes the baseline characteristics of the controls and patients. A total of 89 ischemic stroke patients (mean age, 58.07 ± 9.37 years; 80.90% males) were enrolled in the study. All the patients were caused by symptomatic anterior ICAS. Among these patients, 23 had an sICAS in ICA, 66 in MCA. Eleven had history of ischemic stroke/transient ischemic attack. In one‐way ANOVA/Kruskal–Wallis rank sum test, there were significant differences of body mass index (BMI), C‐reactive protein (CRP), National Institutes of Health Stroke Scale (NIHSS) on admission, antiplatelet drug treatment, stenosis degree, collateral grading, TICI, and 3‐month mRS among patients subgroups. Further multiple comparison showed that patients with penetrating artery disease had significantly lower BMI than other sub‐groups, all *p* < .05. Patients with artery‐to‐artery embolism had significantly lower NIHSS on admission, more favorable 3‐month clinical outcome than other sub‐groups, all *p* < .05. And patients with mixed mechanism had poorer collateral grading than other sub‐groups, all *p* < .05.

**Table 1:** The demographic and clinical characteristics of healthy controls and ischemic stroke patients

Variable	Healthy controls (*n* = 90)	Total patients (*n* = 89)	POPA group (*n* = 29)	A‐A group (*n* = 20)	H group (*n* = 23)	M group (*n* = 17)	*p*‐value
Gender (male/female)	62/28	72/17	25/4	17/3	15/8	15/2	.172
Age (years)	56.38 ± 11.96	58.07 ± 9.37	56.28 ± 9.58	58.45 ± 10.21	60.30 ± 8.14	57.65 ± 9.70	.493
SBP at admission (mmHg)	125.24 ± 17.71	150.63 ± 23.06*	154.56 ± 24.75*	147.20 ± 24.55*	154.59 ± 21.33*	143.29 ± 19.96*	.313
DBP at admission (mmHg)	73.32 ± 11.00	90.93 ± 14.73*	93.85 ± 15.39*	88.40 ± 18.58*	92.50 ± 12.93*	87.24 ± 9.87*	.401
Fast blood glucose (mmol/L)	5.83 ± 1.35	6.89 ± 3.27*	6.05 ± 2.07*	7.20 ± 3.40*	7.72 ± 4.47*	6.48 ± 2.34*	.413
Heart rate (bpm)	68.49 ± 8.94	69.42 ± 12.16	68.38 ± 12.55	70.27 ± 11.94	66.84 ± 9.38	73.35 ± 14.80	.454
BRS	8.05 ± 4.15	6.91 ± 3.76	6.61 ± 3.22	6.96 ± 3.85	7.52 ± 4.25	6.31 ± 3.91	.795
BMI	24.43 ± 2.75	24.19 ± 2.90	25.41 ± 3.05	23.78 ± 3.38	23.90 ± 2.18	23.01 ± 2.27	.036**
Triglycerides (mmol/L)	1.39 ± 0.44	1.68 ± 1.09*	1.73 ± 1.00	1.60 ± 0.86	1.70 ± 1.34	1.67 ± 1.18	.942
HDL (mmol/L)	1.23 ± 0.25	1.00 ± 0.23*	1.05 ± 0.29*	0.99 ± 0.18*	0.98 ± 0.19*	0.96 ± 0.21*	.531
LDL (mmol/L)	2.79 ± 0.56	2.84 ± 0.85	3.14 ± 0.88	2.81 ± 0.74	2.71 ± 0.84	2.60 ± 0.86	.194
CRP (mg/L)	0.74 ± 0.33	5.00 (1.16, 13.17)*	5.00 (1.07, 7.00)*	3.74 (1.25, 17.00)*	5.74 (1.25, 17.00)*	14.00(1.48,15.50)*	.045**
Smoker (%)	0 (0)	41 (46.07)	15 (51.72)	11 (55.00)	8 (34.78)	7 (41.18)	.190
Drinker (%)	0 (0)	8 (8.99)	4 (13.79)	1 (5.00)	2 (8.70)	1 (5.88)	.697
Hypertension (%)	0 (0)	54 (60.67)	19 (65.52)	12 (60.00)	13 (56.52)	10 (58.82)	.923
Diabetes mellitus (%)	0 (0)	26 (29.21)	7 (24.14)	8 (40.00)	7 (30.43)	4 (23.53)	.622
Dyslipidemia (%)	0 (0)	54 (60.67)	18 (62.07)	11 (55.00)	14 (60.87)	11 (64.71)	.938
Prior ischemic stroke or TIA (%)	0 (0)	11 (12.36)	1 (3.45)	5 (25.00)	3 (13.04)	2 (11.76)	.185
Interval from onset to admission (hour)	–	48.00 (24.00, 91.25)	48.00 (15.00,96.00)	24.00 (19.50, 78.00)	48.00 (24.00, 72.00)	72 (28, 132)	.394
Interval from onset to CT/MRI (hour)	–	72.00 (24.00, 120.00)	48.00 (24.00,114.00)	60.00 (23.00, 108.00)	48.00 (16.00, 120.00)	72 (61, 120)	.525
NIHSS on admission	–	3 (1, 8)	3.50 (1.25, 7.75)	1 (0, 2)	6 (1, 10)	3(0.5, 8.5)	.042**
*Antiplatelet drug treatment (%)*	–						
Aspirin	–	57 (64.04)	20 (68.97)	13 (65.00)	16 (69.57)	8 (47.06)	.455
Plavix	–	62 (69.66)	20 (68.97)	16 (80.00)	10 (43.48)	16 (94.12)	.006**
Cilostazol	–	4 (4.49)	1 (3.45)	1 (5.00)	0 (0)	2 (11.76)	.907
*Antihypertension treatment (%)*	–						.095
ARB	–	8 (8.99)	3 (10.34)	0 (0)	2 (8.70)	3 (17.65)	
C	–	25 (28.09)	13 (44.83)	3 (15.00)	6 (26.09)	3 (17.65)	
ARB + C	–	9 (10.11)	3 (10.34)	4 (20.00)	2 (8.70)	0 (0)	
A + ARB+C	–	1 (1.12)	0 (0)	1 (5.00)	0 (0)	0 (0)	
*Side (%)*	–						.845
Left	–	51 (57.30)	18 (62.07)	12 (60.00)	13 (56.52)	8 (47.06)	
Right	–	38 (42.70)	11 (37.93)	8 (40.00)	10 (43.48)	9 (52.94)	
*Large artery stenosis/occlusion (%)*	–						.076
MCA	–	66 (74.16)	29 (100%)	11 (55.00)	14 (60.87)	12 (70.59)	
ICA	–	23 (25.84)	0 (0)	9 (45.00)	9 (39.13)	5 (29.41)	
*Stenosis degree (%)*	–						.075
Moderate stenosis (50%–70%)	–	38 (42.70)	16 (55.17)	11 (55.00)	7 (30.43)	4 (23.53)	
Severe stenosis (70%–99%)	–	29 (32.58)	8 (27.59)	6 (30.00)	6 (26.09)	9 (52.94)	
Occlusion	–	22 (24.72)	5 (17.24)	3 (15.00)	10 (43.48)	4 (23.53)	
*Collateral grading (%)*	–	44 (49.44)	14 (48.28)	9 (45.00)	14 (60.87)	7 (41.18)	.057
Grade 0	–	0 (0)	0 (0)	0 (0)	0 (0)	0 (0)	
Grade 1	–	2 (4.55)	0 (0)	0 (0)	1 (7.14)	1 (14.28)	
Grade 2	–	7 (15.91)	4 (28.57)	0 (0)	0 (0)	3 (42.86)	
Grade 3	–	29 (65.91)	8 (57.14)	6 (66.67)	12 (85.72)	3 (42.86)	
Grade 4	–	6 (13.64)	2 (14.29)	3 (33.33)	1 (7.14)	0 (0)	
*TICI分级 (%)*		51 (57.30)	14 (48.28)	13 (65.00)	15 (65.22)	9 (52.94)	.054
0	–	9 (17.65)	2 (14.29)	2 (15.38)	3 (20.00)	2 (22.22)	
1	–	3 (5.88)	0 (0)	0 (0)	3 (20.00)	0 (0)	
2a	–	8 (15.69)	3 (21.43)	1 (7.69)	1 (6.67)	3 (33.33)	
2b	–	11 (21.57)	0 (0)	5 (38.46)	5 (33.33)	1 (11.11)	
3	–	20 (39.22)	9 (64.29)	5 (38.46)	3 (20.00)	3 (33.33)	
4	–	0 (0)	0 (0)	0 (0)	0 (0)	0 (0)	
*3‐month mRS (%)*	–						.005**
0–2	–	52 (58.43)	17 (58.62)	18 (90.00)	9 (39.13)	8 (47.06)	
3–6	–	37 (41.57)	12 (41.38)	2 (10.00)	14 (60.87)	9 (52.94)	
Recurrent ischemic stroke (%)	–	6 (6.74)	0 (0)	1 (5.00)	3 (13.04)	2 (11.76)	.228

Abbreviations: A, alpha blockers; A‐A, artery‐to‐artery embolism; ARB, Angiotensin Receptor Blocker; BMI, body mass index; C, calcium channel blockers; CRP, C‐reactive protein; CT, Computed Tomography; DBP, diastolic blood pressure; H, hypoperfusion; HDL, High‐density lipoprotein; ICA, internal carotid artery; LDL, Low‐density lipoprotein; M, mixed mechanism; MCA, middle cerebral artery; MRI, Magnetic Resonance Imaging; mRS, modified Rankin Scale; NIHSS, National Institute of Health stroke scale; POPA, parent artery atherosclerosis occluding penetrating artery; SBP, systolic blood pressure; TIA, transient ischemic attack; TICI, Thrombolysis In Cerebral Infarction.

*p*‐value: *p*‐value of comparing among stroke patients subgroup.

* Significant difference in comparing with control.

** Significant difference in comparison among stroke patients subgroups.

Ninety healthy volunteers (56.38 ± 11.96 years; 69.66% males) served as controls. Comparing with patients, healthy controls presented with significantly lower blood pressure, lower fast blood glucose, CRP, TG, and higher HDL, all *p* < .05. Table 1.

### 4.2 Cerebral autoregulation in different stroke mechanisms

To assess the intrarater reproducibility of the classification criteria, 1 investigator (Z.Z.Lin) performed stroke mechanisms classification in all cases twice, with at least 30 days apart in the 2 assessments in each case. To assess the interrater reproducibility, 2 investigators (Z.Ji and Z.Z.Lin) independently performed image assessment in all cases. The intrarater reproducibility was excellent (κ, 0.848; 95% CI, 0.760–0.936), and the interrater reproducibility was substantial (κ, 0.774; 95% CI, 0.672–0.876), when classifying the stroke mechanisms into 4 categories.

In the control group of healthy volunteers, the phase difference (PD; 0.06–0.12) between the arterial blood pressure (BP) and cerebral blood flow velocity (CBFV) was 54.51 ± 21.83 degrees in the left hemisphere and 54.76 ± 21.96 degrees in the right hemisphere, and these values were not significantly different (*p* = .249). The overall PD in this group was 54.64 ± 21.87 degrees.

PD in ischemic stroke patients was significantly lower than healthy controls bilaterally, (54.64 ± 21.87 versus 37.02 ± 21.73, 54.64 ± 21.87 versus 26.78 ± 21.25, all *p* < .001).

**Figure 5:**
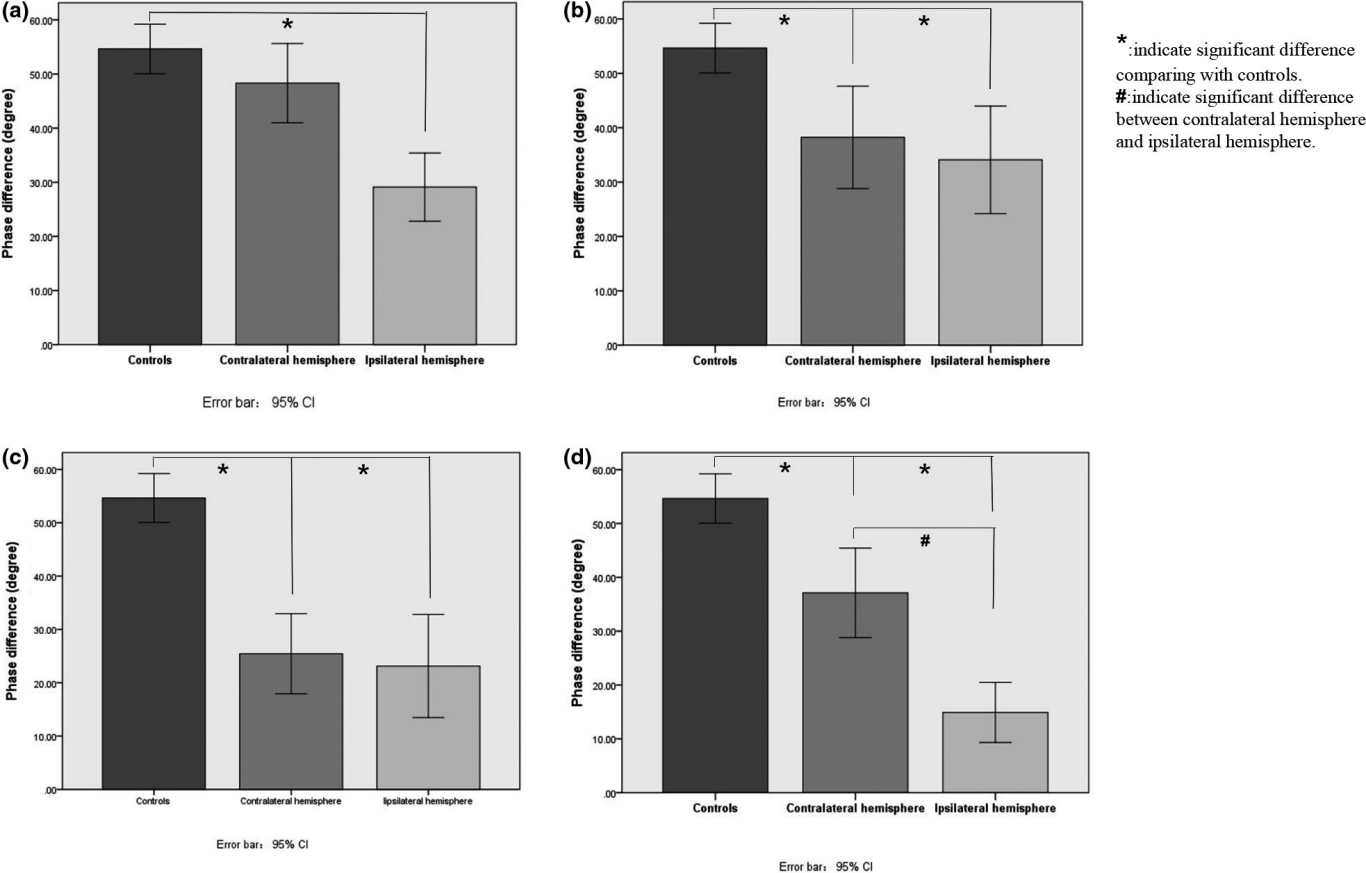
(a) Compared with controls, CA was impaired ipsilaterally in ischemic stroke patients with parent artery atherosclerosis occluding penetrating artery (POPA). (b) Compared with controls, CA was impaired bilaterally in ischemic stroke patients with Artery‐to‐Artery embolism stroke mechanism. And CA was similar between two hemispheres in ischemic stroke patients. (c) Compared with controls, CA was impaired bilaterally in ischemic stroke patients with hypoperfusion stroke mechanism. And CA was similar between two hemispheres in ischemic stroke patients. (d) Compared with controls, CA was impaired bilaterally in ischemic stroke patients with mixed pattern stroke mechanism. And CA was lower on the ipsilateral hemisphere than that on the contralateral hemisphere in ischemic stroke patients

In patients with parent artery atherosclerosis occluding penetrating artery, the PD in the middle cerebral arteries of the ipsilateral hemisphere was significantly lower than controls (29.11 ± 16.56 versus 54.64 ± 21.87, *p* < .001) and also was significantly lower than contralateral hemisphere (29.11 ± 16.56 versus 48.30 ± 19.25, *p* < .001). No significantly difference of PD was observed between controls and unaffected hemisphere, *p* > .05, (Figure 5a).

In the artery‐to‐artery embolism group, PD on bilateral hemispheres was similar (38.22 ± 20.13 versus 34.09 ± 21.13, *p* = .457) and was both significantly lower than controls, all *p* < .05, (Figure 5b).

In patients with ischemic stroke caused by hypoperfusion, PD on both ipsilateral hemisphere and contralateral hemisphere was similar (25.45 ± 17.37 versus 23.13 ± 22.34, *p* = .628) and was significantly decreased comparing with healthy controls, all *p* < .05, (Figure 5c).

Bilaterally, the PD was significantly lower in patients with mixed mechanisms than controls, all *p* < .05, and PD on ipsilateral hemisphere was significantly lower than on contralateral hemisphere, 14.91 ± 10.86 versus 37.11 ± 16.16, *p* < .001, (Figure 5d).

### 4.3 Correlation between cerebral autoregulation and clinical parameters/collateral circulation

Simple linear regression analysis was performed to assess the correlation between cerebral autoregulation and clinical parameters. Then, we performed multiple regression analysis with adjusting confounding factors including age, sex, BP, low‐density lipoprotein (LDL), CRP in different subgroups.

In patients with parent artery atherosclerosis occluding penetrating artery, the correlation between phase on ipsilateral hemisphere and history of hypertension/hyperlipidemia was favorable respectively, (*r* = −0.481, *p* = .008; *r* = −0.484, *p* = .008). The multiple regression analysis showed that phase on ipsilateral hemisphere had relationship with hypertension/hyperlipidemia, (*p* = .059, *p* = .050, respectively).

**Figure 6:**
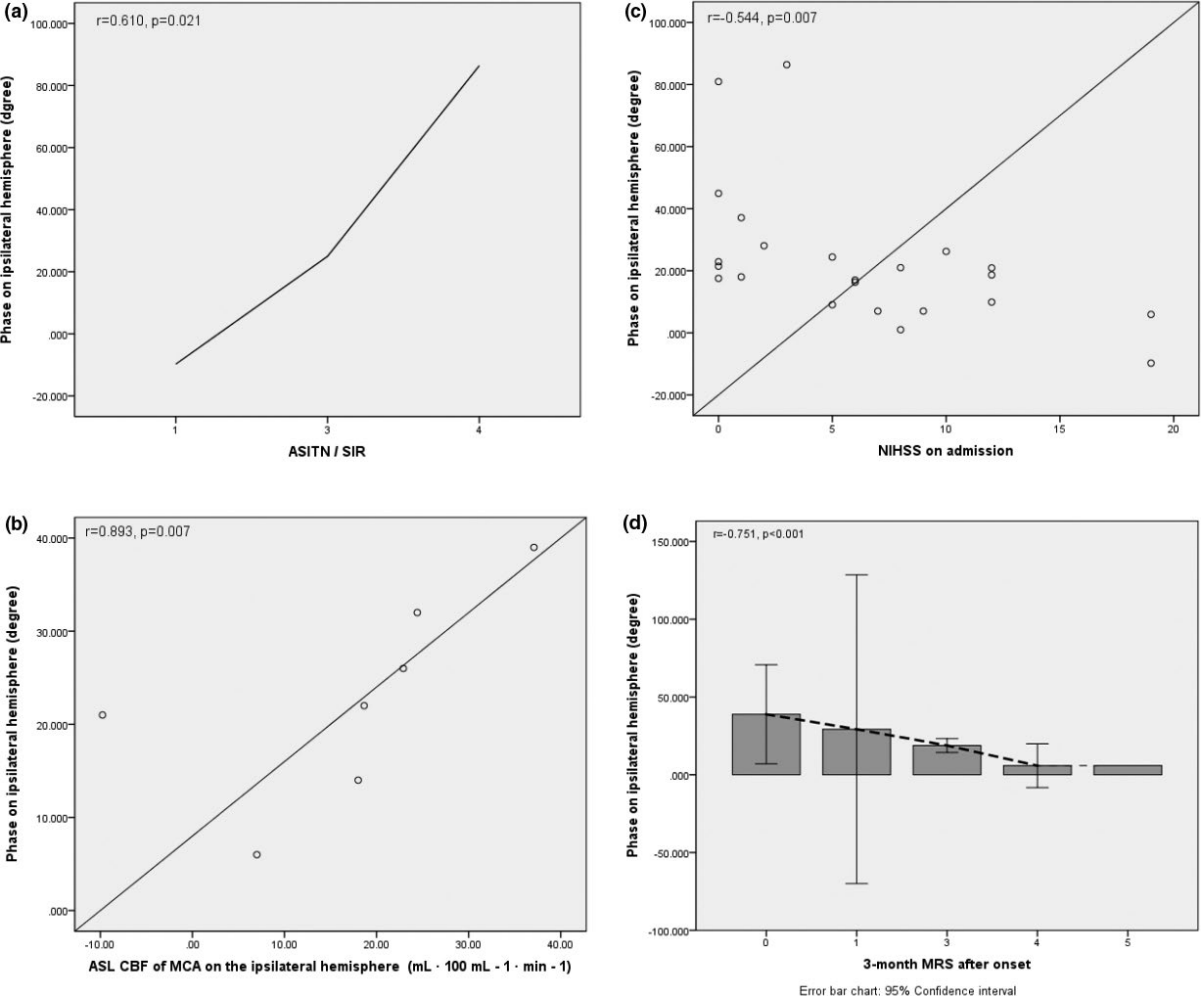
(a) In patients with ischemic stroke caused by hypoperfusion, there was an excellent correlation between the PD on ipsilateral hemisphere and ASITN/SIR collateral status. (b) In assessing the relationship between PD on ipsilateral hemisphere and arterial spin‐labeling parameter CBF, significantly correlation was observed in patients with ischemic stroke caused by hypoperfusion. (c) In patients with ischemic stroke caused by hypoperfusion, NIHSS on admission was significantly correlated with PD on ipsilateral hemisphere in the subgroup. (d) In patients with ischemic stroke caused by hypoperfusion, clinical outcome on 3‐month after ischemic stroke was significantly correlated with PD on ipsilateral hemisphere in the subgroup

In patients with ischemic stroke caused by hypoperfusion, there was an excellent correlation between the PD on ipsilateral hemisphere and ASITN/SIR collateral status (*r* = 0.610, *p* = .021). The multiple regression analysis showed that phase on ipsilateral hemisphere was still significantly correlated with the ASITN/SIR collateral status, *p* = .015. (Figure 6a). In assessing the relationship between PD on ipsilateral hemisphere and ASL parameter CBF, significantly correlation was observed, (*r* = 0.893, *p* = .007), and the multiple regression analysis showed that phase on ipsilateral hemisphere was correlated with ASL parameter CBF, *p* = .039. (Figure 6b).

For clinical outcome, we found that both NIHSS on admission and 3‐month mRS were significantly correlated with PD on ipsilateral hemisphere in the subgroup caused by hypoperfusion, (*r* = −0.544, *p* = .007; *r* = −0.751, *p* < .001; Figure 6c,d).

No other significant correlation was observed in other subgroups, all *p* > .05.

## 5 DISCUSSION

In the study, we found that (a) CA was impaired bilaterally in IS patients caused by ICAS and (b) the CA impairment pattern was heterogeneous in different stroke mechanism caused by ICAS. In patients with POPA, CA was only impaired on ipsilateral hemisphere and the decrease of CA was correlated with hypertension/hyperlipidemia history. In patients with hypoperfusion mechanism, CA was impaired bilaterally, a liner association was found between CA impairment and collateral status/perfusion parameters. In A‐A embolism and mixed mechanism subgroups, CA was impaired bilaterally, but no significant correlation was observed between CA and any factors. All findings above agree with our hypothesis and suggest that CA impairment pattern is varied in different stroke mechanism in patients with IS caused by ICAS. Moreover, the dysfunction pattern of CA was correlated with different risk factors in different stroke mechanisms.

As mentioned by previous studies, ICAS has been shown to be an independent risk factor for subsequent ischemic stroke (Wong, 2006). And the latest treatment is from the perspective of improving hemodynamic situation.

Recently, numerous researches indicated that the evaluation of the functional reserve of cerebral hemodynamics in patients with ICAS should become an integral part of the routine examination in order to optimize the treatment strategy. Under normal condition, autoregulatory cerebral vasodilatation occurs to maintain the CBF against the effect of hypoperfusion. When vasomotor compensatory mechanisms fail, the hemodynamics stable will be impaired. In our study, we observed significantly lower CA on both the affected and unaffected sides in most IS patients with ICAS except patients caused by POPA, comparing with healthy controls. Our finding was partly consist with previous reports, since debates existed about the pattern of CA impairment in ischemic stroke patients. Data from previous studies showed that CA was more likely impaired focally on the affected side in acute unilateral IS with LAA (Guo et al., 2014; White & Markus, 1997). While some studies showed that CA was bilaterally impaired in both the affected and nonaffected hemispheres (Dawson et al., 2003). We hypothesis that the debates may be attributed to different stroke mechanism in ICAS. In our study, we observed that CA was only impaired on affected side in patients with POPA, while CA was impaired bilaterally in other stroke mechanisms of ICAS. To further analyze the potential reason of the different pattern, we performed correlation analysis of CA and detail factors in different stroke mechanism subgroups.

Liner association analysis showed that CA on ipsilateral hemisphere was closely correlated with history of hypertension/hyperlipidemia in patients with POPA. Partly consisting with our finding, Kim et all reported that in patients with local branch occlusion, ICAS was most frequently associated with the hypertension, and in patients with anterior circulation diseases, external carotid artery stenosis was more closely associated with hyperlipidemia (Kim et al., 2012). To our knowledge, the primary mechanism of IS that caused by atherosclerotic disease of MCA is plaque rupture, which leads to occlusion of small penetrating arteries (Zhao et al., 2016). Hypertension and hyperlipidemia are two major factors affect plaque stability. Under the pathological microenvironment, local hemodynamic status may be influenced and further affect stroke improvement (Rothwell et al., 2000). Whatever the explanation, our data seem to suggest that the decrease of CA was more likely correlated to atherosclerosis risk factors, such as hypertension or hyperlipidemia in IS patients with POPA mechanism. Moreover, due to the formation mechanism of POPA which was mentioned above, hemodynamics disorder and vascular structure damage just occurred on the ipsilateral hemisphere. Therefore, CA was just unilateral damage in the POPA subtype.

In patients with hypoperfusion mechanism, we found that CA was impaired bilaterally. And the impairment of CA was significantly correlated with perfusion parameters/collateral circulation status. As we know, under normal physiologic conditions, CA maintains stable cerebral perfusion (Greene & Lee, 2012).

But in stroke patients with ICAS, CA was impaired and further leading to perfusion disorder. Basing on the pathophysiology situation, if a patient's anatomy does not permit adequate collateral flow, hypoperfusion region of the brain will enlarge and progress to infarct. Meanwhile, infarct‐related changes of microenvironmental including cerebral oxygen metabolism affect endothelial function and further deteriorate cerebral dysfunction (Anderson, 1999). In addition, as demonstrated by previous studies that reduced cerebrovascular reserve capability, including CA associated with the anatomic characteristics of intracranial collateralization (Berkhemer et al., 2016; Vernieri et al., 2001). Moreover, as we known, infarction often occurred unilaterally. We hypothesis that it may be attributed to the local artery condition, including artery stenosis degree, shear stress, plaque characteristics, local hemodynamics, and so on. With the development of vascular imaging technology, further research is needed to be performed. In generally, CA, hypoperfusion, and insufficient collateral circulation may act alone and complement interactively, causing ischemic stroke in patients with ICAS (Wong et al., 2016). In addition, experimental studies have shown that permanent distal occlusion of the MCA in rat brain increased the contractile response to angiotensin, 5‐hydroxytryptamine, and endothelin in segments downstream of the occlusion (Rasmussen et al., 2013). And Coucha et al, demonstrated that ischemic injury impaired the vascular reactivity of rat MCAs in the ischemic and nonischemic hemispheres via increased peroxynitrite generation after stroke (Coucha et al., 2013). It may indicate that some chemicals will be produced during the hypoperfusion process, even transfer to the contralateral side to further affect CA bilaterally. However, further research both of laboratory research and clinical trail is needed in future.

We also observed a close association of CA and 3‐month outcome in stroke patients with hypoperfusion mechanism, which agree with previous studies showing that there is a strong dependence on the poor‐outcome of ischemic stroke in patients with occlusive disease producing hypoperfusion. Early studies also revealed that the evaluation of collateral flow and hemodynamic status may offer better prognostication regarding stroke recurrence (Berkhemer et al., 2016; Wouters et al., 2016). Nowadays, the major therapeutic strategies for ICAS contain: (a) antithrombotics, (b) intervention to prevent thromboembolism and restore blood flow, and (c) identification and control of risk factors (Bang, 2014), and treatment effects differ among the different types of ICAS (Jung et al., 2009). Although several studies including ours have strongly suggested that the risk for IS was higher in patients with impaired CA, cerebral perfusion and hemodynamic reserve capacity were still not be considered as guidelines for the treatment of ICAS. Assessment of CA should therefore be used in future treatment planning making in individual patients.

Although our study is a clinical CA study with innovative perspective to discuss the CA of different stroke mechanism caused by ICAS, which may provide a deeper understanding of CA and physiopathology of different stroke mechanism, there are still some limitations. Firstly, since this is a primarily observational study confined by small sample size of subgroups, although CA of various subgroups of ischemic stroke patients were studied, only limited information could be inferred with the current data for correlation analysis could not be provided any causal relationship. In further study, large sample size studies are needed. Secondly, we recruited more male subjects than female in our study, since there are more male stroke patients than female in China (Liu et al., 2007), and female subjects are more likely to have insufficient bilateral temporal bone windows for insonation due to the low density of the temporal bone (Wijnhoud et al., 2008).

In conclusion, our study suggests that CA is heterogeneous impaired in ischemic stroke patients caused by ICAS with different stroke mechanism. And CA should be assessed in patients with different stroke mechanism caused by ICAS.

## CONFLICTS OF INTEREST

The authors declare that there are no conflicts of interest regarding the publication of this article.

## AUTHOR CONTRIBUTION

Tian G involved in study concept and design, acquisition of data, analysis and interpretation of data, drafting the manuscript, and revision the manuscript for content. Ji Z involved in analysis and interpretation of data, revision of the manuscript for content. Lin ZZ involved in acquisition and analysis of data, and image analyze. Pan SY. involved in supervision of analysis and revision of the manuscript for content. Yin J. involved in study concept and design, critical revision of manuscript, and study supervision.

### Peer Review

The peer review history for this article is available at https://publons.com/publon/10.1002/brb3.1907.

## Data Availability

The raw/processed data required to reproduce these findings cannot be shared at this time as the data also forms part of an ongoing study.
